# Analysis of Pericoronary Adipose Tissue Attenuation in Patients with Type 2 Diabetes Mellitus on Angiotensin-Converting Enzyme Inhibitors and Angiotensin Receptor Blockers: A Propensity-Score-Matched Observational Study

**DOI:** 10.3390/biomedicines14061268

**Published:** 2026-06-02

**Authors:** Bryan Wu, Hanyi Joh, Koen Nieman, Ryan Sandoval

**Affiliations:** 1 Department of Internal Medicine, Division of Cardiovascular Medicine, Stanford University, Stanford, CA 94305, USA; 2 Department of Molecular Biology, Pomona College, Claremont, CA 91711, USA

**Keywords:** type 2 diabetes mellitus, vascular inflammation, pericoronary adipose tissue attenuation, renin-angiotensin–aldosterone system inhibitors

## Abstract

**Background:** In patients with type 2 diabetes mellitus (T2DM), angiotensin-converting enzyme inhibitors (ACE-Is) and angiotensin receptor blockers (ARBs) are first-line antihypertensive treatments with important cardiovascular benefits, but their impacts on coronary-specific inflammation are unknown. Pericoronary adipose tissue (PCAT) attenuation, as assessed by coronary computed tomography angiography (CCTA), serves as a specific biomarker for coronary inflammation. Here, we aim to assess whether treatment with ACE-I or ARB is correlated with lower PCAT attenuation. **Methods:** In this retrospective observational study, we analyzed 223 patients with T2DM and coronary atherosclerosis who underwent CCTA from 1 January 2017 to 1 September 2024 at our institution. PCAT attenuation was measured in the proximal right coronary artery. Propensity score matching and multivariate linear regression analyses were performed for comparisons. **Results:** Of the 223 patients (mean age of 64.9 ± 8.8 years, 69.1% male), 122 patients were on ACE-I or ARB (ACE-I/ARB). ACE-I/ARB users had similar PCAT attenuation as their counterparts after propensity score matching (−72.1 ± 7.5 and −71.7 ± 8.1 HU, respectively; *p* = 0.722). Subgroup analysis in patients with glomerular filtration rate (GFR) < 90 mL/min revealed lower PCAT attenuation in ACE-I/ARB users (−74.8 ± 6.6 vs. −71.4 ± 7.1 HU; *p* = 0.038), with a significant interaction between these two factors in the multivariate analysis (*p* = 0.047). Other antihypertensive treatments (beta blockers, dihydropyridine calcium channel blockers, and thiazides) were not linked with lower coronary inflammation. **Conclusions:** In T2DM patients with coronary atherosclerosis, we did not find an association between ACE-I/ARB treatment and lower coronary inflammation as defined by PCAT attenuation, although such a relationship may exist in those with reduced GFRs.

## 1. Introduction

Angiotensin receptor blockers (ARBs) and angiotensin-converting enzyme inhibitors (ACE-Is) are first-line antihypertensive therapies for patients with type 2 diabetes (T2DM), and several studies have demonstrated the cardiovascular benefits linked with renin–angiotensin–aldosterone system (RAAS) inhibitors in this high-risk cohort [1,2]. Earlier studies have shown that angiotensin II can lead to a higher production of proinflammatory cytokines, reactive oxygen species, and adhesion molecules [3,4]. Its involvement in coronary atherogenesis has been suggested, as patients with critical coronary artery disease have higher plasma levels of angiotensin II [5]. ACE-Is and ARBs have demonstrated anti-inflammatory potential via reductions in systemic circulatory biomarkers, such as C-reactive proteins and interleukin-6 [6]. However, it remains unknown whether ACE-I and ARB treatments are linked to lower inflammatory activity that is more specific to the coronary artery system.

Pericoronary adipose tissue (PCAT) attenuation is a novel imaging biomarker for coronary inflammation, which is associated with plaque progression and rupture. As assessed by coronary computed tomography angiography (CCTA), PCAT is the epicardial adipose tissue that is directly adjacent to the coronary vessel. Heightened inflammation from the coronary plaque impairs the maturation of adipocytes and reduces their size [7]. This intricate process ultimately leads to an elevated density of adipocytes of the PCAT, which corresponds to higher Hounsfield units (HU) on CCTA. A mean PCAT attenuation above −70.5 HU in the proximal right coronary artery (RCA), the traditional site of assessment, is predictive of future myocardial infarction with a hazard ratio of 2.45 [8]. This biomarker has been shown to outperform CT calcium score, cardiovascular risk score, and even the presence of obstructive lesions in predicting future myocardial infarction [8].

T2DM and vascular inflammation are closely linked, as hyperglycemia promotes the increased production of reactive oxygen species and the secretion of pro-inflammatory cytokines [9]. In this retrospective analysis, we aim to evaluate whether treatment with an ACE-I or ARB is correlated with lower coronary inflammation in this high-risk cohort. We chose to assess PCAT attenuation because it reflects the localized inflammation within the coronary artery system. This biomarker has demonstrated superiority in coronary risk stratification compared with commonly used circulating biomarkers, such as C-reactive protein, interleukin-6, and tumor necrosis factor-α [10]. Yu et al. found that patients with T2DM have higher PCAT attenuation than those without T2DM, independent of the presence of obstructive stenoses [11]. While high-intensity statin and glucagon-like peptide-1 receptor agonist treatments are associated with lower inflammatory activity, as measured by this novel imaging biomarker [12,13,14], the impacts of other pharmacological therapies have not been well characterized in patients with and without T2DM. To our knowledge, there has been no study that closely assessed the effects of antihypertensive therapies on PCAT attenuation in patients with coronary atherosclerosis.

## 2. Materials and Methods

### 2.1. Patient Screening and Clinical Data Collection

In this retrospective observational study, we screened patients who underwent CCTA from 1 January 2017 to 1 September 2024 at Stanford Health Care. To qualify for the diagnosis of T2DM and hypertension, patients must have had the diagnosis clearly documented in a physician’s note. Additionally, for T2DM, patients needed to have prior fasting glucose ≥ 126 mg/dL, hemoglobin A1C (HbA1C) ≥ 6.5%, random glucose ≥ 200 mg/dL, or a blood glucose level of ≥200 mg/dL on a 2-h oral glucose tolerance test. For hypertension, patients must either have received antihypertensive treatment for ≥6 months or have a documented blood pressure ≥ 130/80 mmHg during two or more clinic visits. The inclusion criteria were as follows: aged 40–80 years; diagnosis of T2DM with most recent HbA1C ≤ 9.0%; body mass index (BMI) ≤ 35.0; and evidence of coronary atherosclerosis on CCTA (as defined by Coronary Artery Disease-Reporting and Data System [CAD-RADS] grading ≥1); computed tomography (CT) tube voltage 100–120 kV; and treatment with at least 1 oral antidiabetic agent for ≥6 months. We excluded patients who had suboptimal imaging quality, prior coronary interventions, and anomalous coronary anatomy, including the non-dominant right coronary artery. The majority of our patients had not previously undergone coronary imaging. We also excluded patients with obstructive lesions in the right coronary artery, as these findings could raise PCAT attenuation in that region and thus misrepresent global inflammation for the entire coronary system.

We collected patients’ demographic information, atherosclerotic risk factors, and medication history. Patients’ medication regimens were verified for analysis if the treatment duration was greater than 6 months. Additionally, we recorded patients’ blood pressures at the time of CCTA acquisition (before beta blockers and nitroglycerin were administered) and during the two outpatient clinic encounters within 6 months prior to scan acquisition, if available. Laboratory data, including lipid panel and basic metabolic panel, were also documented. The glomerular filtration rate was calculated using the 2021 Chronic Kidney Disease Epidemiology Collaboration (CKD-EPI) equation using sex, age, and serum creatinine.

### 2.2. CT Imaging Acquisition and PCAT Analysis

**Figure 1:**
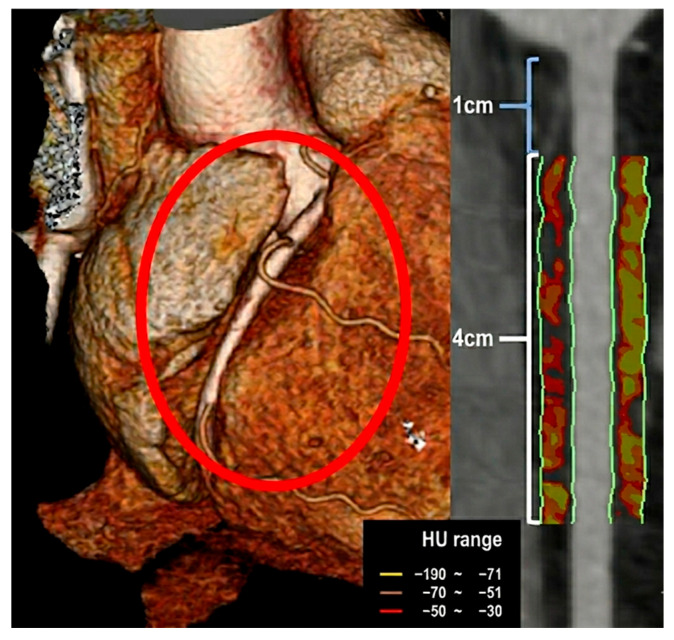
Illustration of a patient’s pericoronary adipose in the proximal right coronary artery (1 cm away from the origin, along a 4 cm length; the width is defined by the diameter of the vessel). Red indicates regions of higher radiodensity or more inflamed adipose tissue. Acronym: HU, Hounsfield unit.

The CCTAs were acquired on a third-generation, dual-source, 384-slice (2 × 192) CT scanner (Siemens Somatom FORCE, Forchheim, Germany) with retrospective EKG-gating. Patients were instructed to hold their breath during the image acquisition. Oral beta blockers were administered if their resting heart rate exceeded 70 beats per minute, and sublingual nitroglycerin was given to all patients to allow for coronary dilatation. The severity of coronary artery disease, as defined by the CAD-RADS classification system, and coronary artery calcium (CAC) scores were recorded. The PCAT analysis was carried out using the Terarecon Aquarius Intuition 4.4.13 software by a cardiologist who is board-certified in cardiac CT and blinded to patients’ clinical profiles. For the majority of the scans, end-diastolic phase images were used for analyses. The PCAT attenuation was measured in the proximal RCA, starting from 1 cm distal to the coronary origin along a 4-cm length, and the width is defined as the diameter of the coronary vessel (Figure 1). While there is no universal definition for where PCAT attenuation should be measured, this site is commonly used due to its lack of side branches and abundance of adipose tissue [7]. Within this region of interest, PCAT attenuation is defined as the mean HU attenuation within the range for adipose tissue on CT (−190 to −30 HU). As previously described, PCAT attenuation was divided by a correction factor of 1.11485 for CCTAs acquired with a tube voltage of 100 kV [8].

### 2.3. Statistical Analysis

The IBM SPSS Statistics 30.0.0 software was used for statistical analysis. Two treatment groups were defined based on whether patients were on an ACE-I or ARB. Continuous variables are presented as mean ± standard deviation, while categorical variables are presented as percentages of patients. Univariate analyses were conducted with an unpaired Student’s *t*-test for continuous variables (Mann-Whitney U test for variables with skewed distribution) and either a chi-square test or Fisher’s exact test for categorical variables. Propensity score matching was performed for our analysis. The propensity score was calculated using a logistic regression model accounting for the following: age, sex, BMI, statin intensity, hyperlipidemia, hypertension, presence of obstructive coronary lesions requiring revascularization, CAC score, and current smoking status. Patients from the two treatment groups were matched in a 1:1 ratio using the nearest neighbor method without replacement. The caliper width was defined as 0.2 multiplied by the standard deviation of the logit of the propensity score. Multivariate linear regression analysis was also performed as specified in Section 3, adjusting for the same variables as outlined for the propensity score-matching analysis. For our subgroup analyses, the cutoffs for HbA1C, GFR, and blood pressure were determined based on clinical relevance and evenness in the distribution of patients.

This study was reviewed and approved by the Stanford University Institutional Review Board, and informed consent was waived. Some components of the Graphical Abstract were generated using Google Gemini 3.5.

## 3. Results

**Figure 2:**
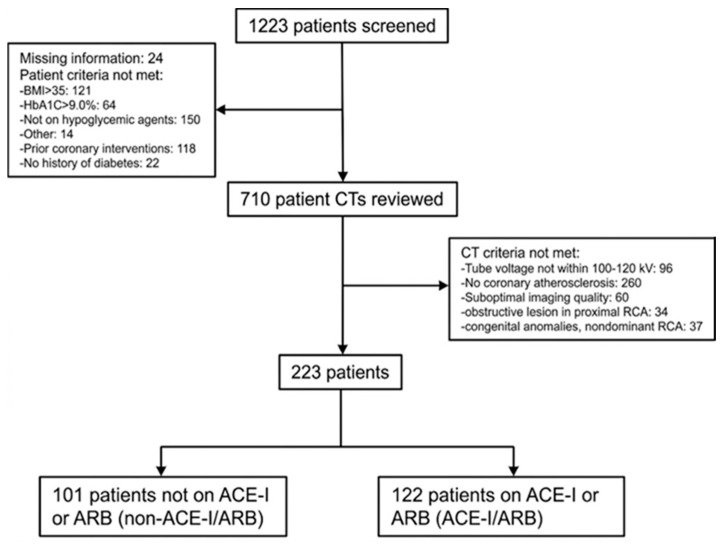
Diagram of patient screening and selection process. Acronyms: BMI, body mass index; HbA1C, hemoglobin A1C; ACE-I, angiotensin-converting enzyme inhibitors; ARB, angiotensin receptor blockers; RCA, right coronary artery; CT, computed tomography.

**Figure 3:**
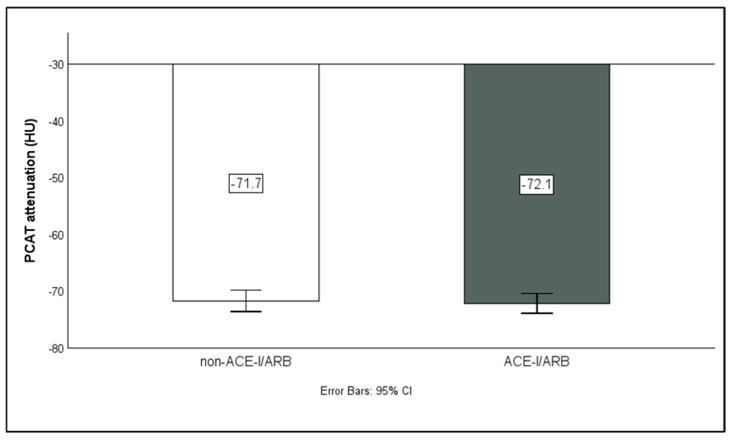
Pericoronary adipose tissue (PCAT) attenuation was similar between patients on angiotensin-converting enzyme inhibitors or angiotensin receptor blockers (ACE-I/ARB) and those not on either treatment (non-ACE-I/ARB) after propensity score matching. Higher PCAT attenuation confers greater coronary inflammation. Acronyms: HU: Hounsfield unit.

As illustrated in Figure 2, a total of 1223 patients were screened, of whom 223 patients met our study criteria and were included in the study. In total, 122 patients were on either an ACE-I (*n* = 55) or ARB (*n* = 67), while 101 patients were not on these antihypertensive therapies. The baseline characteristics are outlined in Table 1. The mean age for our entire study cohort was 64.9 ± 8.8, and the majority of our patients were male (69.1%). Patients in the ACE-I or ARB (ACE-I/ARB) group had a higher prevalence of hypertension (97.5% vs. 75.2%; *p* < 0.001) and hyperlipidemia (92.6% vs. 82.2%; *p* = 0.023), compared to those not on treatment (non-ACE-I/ARB). We observed a right-skewed distribution with respect to the CAC score, and patients on ACE-I/ARB had a non-significantly higher CAC score than non-ACE-I/ARB users (759.5 ± 1026.1 [median: 410.5; interquartile range: 87.0–1004.5] vs. 518.8 ± 781.3 [median: 198.0; interquartile range: 67.0–639.0], respectively; *p* = 0.057). Chest pain evaluation was the common indication for both groups (ACE-I/ARB: 67.2%, non-ACE-I/ARB: 57.4%; *p* = 0.164). The ACE-I/ARB group had a higher proportion of patients on high-intensity statin therapy, which is known to stabilize coronary plaques and reduce inflammation (44.3% vs. 23.8%, *p* = 0.001). Our propensity score matching and multivariable linear regression analyses accounted for age, sex, BMI, statin intensity, hyperlipidemia, hypertension, presence of obstructive coronary lesions requiring revascularization, CAC score, and current smoking status. Male sex and statin intensity were found to be independent predictors of PCAT attenuation (Table 2), which is in concordance with findings from previous studies [8,14]. In our propensity-score-matched analysis, a total of 146 patients were evaluated, and no significant difference in PCAT attenuation was found between ACE-I/ARB users and non-users (−72.1 ± 7.5 and −71.7 ± 8.1 HU, respectively; *p* = 0.722; Table 3 and Figure 3). Furthermore, we performed another propensity-score-matched comparison between patients on the maximal dosage of an ACE-I/ARB (*n* = 44) and non-ACE-I/ARB users (*n* = 44) and found no differences (−74.0 HU ± 7.1 and −73.8 ± 7.9 HU, respectively; *p* = 0.891; Table S1).

**Figure 4:**
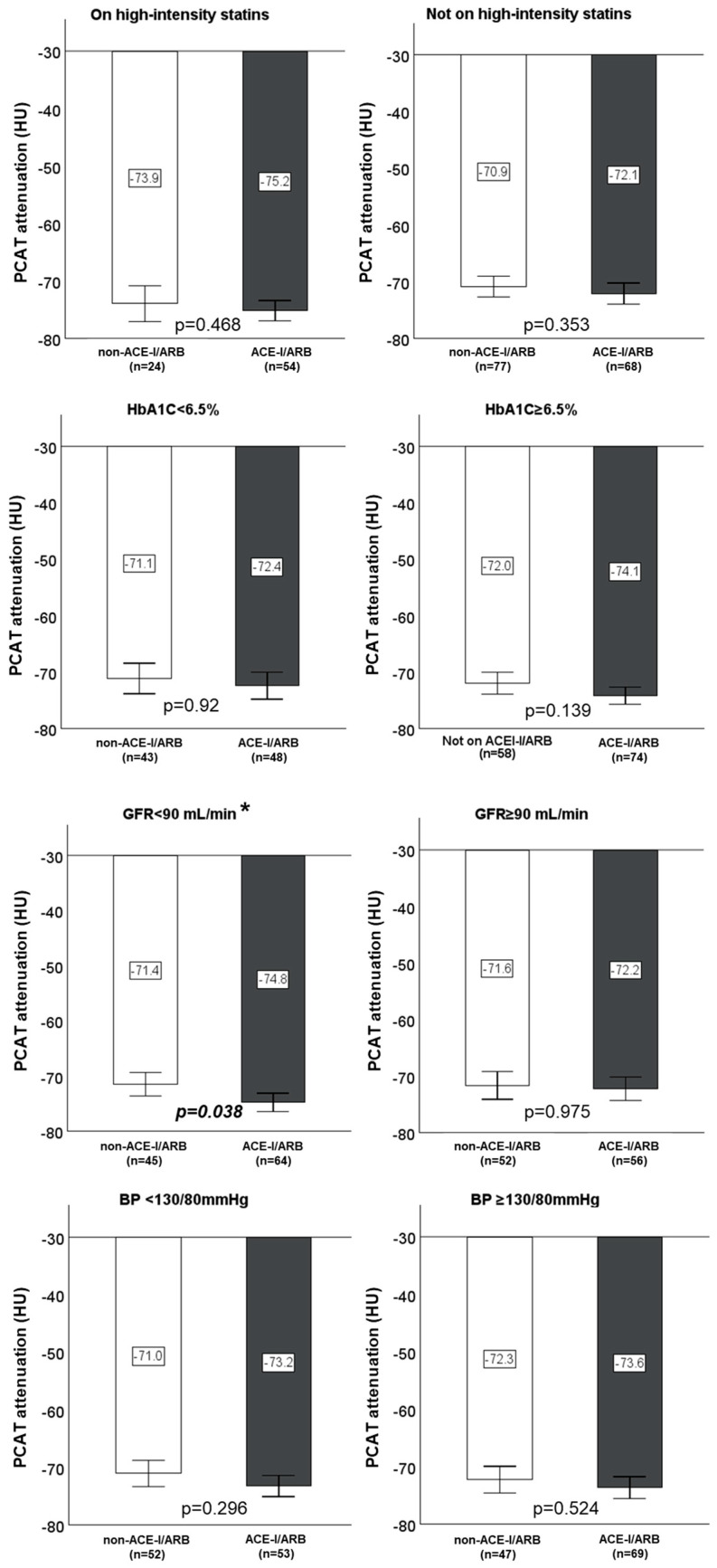
Subgroup analyses of PCAT attenuation in patients on either angiotensin-converting enzyme inhibitors or angiotensin receptor blockers (ACE-I/ARB) versus their counterparts (non-ACE-I/ARB). Higher PCAT attenuation confers greater coronary inflammation. Comparisons were made with adjustments to high-intensity statin treatment except for the first subgroup. * Among patients with a glomerular filtration rate (GFR) < 90 mL/min, patients on an ACE-I/ARB had lower pericoronary adipose tissue (PCAT) attenuation. Acronyms: HbA1C, hemoglobin A1C; HU, Hounsfield unit.

For our subgroup analyses (Figure 4), comparisons were carried out with adjustments for high-intensity statin treatment, which has been shown to yield plaque stabilization and has a strong correlation with lower coronary inflammation [12,14]. In total, 97% of patients had their creatinine and GFR assessed in the 6 months prior to CCTA. In patients with GFR < 90 mL/min (*n* = 109), the ACE-I/ARB group (*n* = 46) had lower PCAT attenuation (−74.8 ± 6.6 HU vs. −71.4 ± 7.1 HU; *p* = 0.038). In our multivariate linear regression model, we observed a significant interaction between ACE-I/ARB treatment and the presence of renal impairment with GFR < 90 mL/min in predicting PCAT attenuation (*p* = 0.047). In this subgroup of patients with GFR < 90 mL/min, we also assessed the effect of sodium-glucose cotransporter-2 (SGLT-2) inhibitors (*n* = 28), another class of medications with renal protective properties, but found no difference (SGLT-2 inhibitors: −73.5 ± 6.4 HU; not on SGLT-2 inhibitors: −73.4 ± 7.2 HU; *p* = 0.997). We had a small number of patients with GFR < 60 mL/min (*n* = 24) and noted a non-significant trend towards lower coronary inflammation in ACE-I/ARB users (−76.6 ± 7.4 HU vs. −70.9 ± 6.7 HU; *p* = 0.061). Finally, among patients already on high-intensity statins, ACE-I/ARB users did not have lower PCAT attenuation.

We also performed multivariate linear regression analyses for three other common classes of antihypertensive therapy (dihydropyridine calcium channel blockers, beta blockers, and thiazides) and found no significant association with lower PCAT attenuation (Table 4). Using PCAT attenuation > −70.5 HU as the cutoff, as previously described [8], patients with elevated and non-elevated coronary inflammation were found to be on a similar number of antihypertensive therapies (1.2 ± 1.1 vs. 1.4 ± 1.0, respectively; *p* = 0.156). Furthermore, these two groups had similar systolic blood pressure (elevated PCAT: 131 ± 14 mmHg; non-elevated PCAT: 130 ± 14 mmHg; *p* = 0.875), and patients with elevated coronary inflammation had non-significantly higher diastolic blood pressure (76 ± 28 mmHg vs. 72 ± 11 mmHg; *p* = 0.118).

## 4. Discussion

To our knowledge, this is the first study that evaluated the impact of ACE-Is and ARBs on coronary inflammation according to PCAT attenuation in patients with T2DM, who are at high risk for coronary atherosclerosis. Based on our propensity-score-matched analysis, we did not observe a correlation between treatment with an ACE-I or ARB for more than 6 months and lower PCAT attenuation on CCTA. This lack of association was seen even in patients receiving maximal doses of these medications.

While RAAS inhibitors have been shown to lower serum biomarkers for inflammation [6], evidence supporting their prevention of future myocardial infarction through the stabilization of coronary plaque progression and vulnerability is less robust. In a recent retrospective study by Zhang et al. involving 2501 patients with hypertension, diabetes, and obstructive coronary artery disease on coronary angiogram, those who were on an ACE-I/ARB had lower composite major adverse cardiac and cerebral events but did not have lower incidences of non-fatal MI [15]. In the Quinapril Ischemic Event trial, treatment with quinapril did not reduce ischemic events and had no significant impact on angiographic progression of coronary plaques [16]. By contrast, in the OLIVUS trial, treatment with Olmesartan was shown to yield slower plaque progression, as assessed via intravascular ultrasound, in nonculprit vessels in patients undergoing percutaneous coronary intervention [17]. Nevertheless, a more recent sub-analysis of the PARADIGM registry showed that ACE-Is and ARBs had no significant longitudinal effects on plaque volume and composition [18]. Thus, the plaque-stabilizing potential of RAAS inhibitors is not yet well established.

In our subgroup analyses, we found that among patients with a GFR lower than 90 mL/min, ACE-I/ARB users had lower PCAT attenuation, and there was a significant interaction between these two factors. Chronic kidney disease (CKD) is increasingly recognized as a systemic inflammatory condition characterized by endothelial dysfunction and immune dysregulation, which elevates the risk of accelerated progression of coronary atherosclerosis [19]. Further research is warranted to assess pharmacological strategies for the reduction in long-term cardiovascular and inflammatory risks within this vulnerable cohort. When renal perfusion and GFR are reduced, there is an amplified RAAS activity from an increase in renin production by juxtaglomerular cells that line the afferent arterioles [20]. The increased production of angiotensin II has been shown to induce vascular remodeling through promotion of endothelial dysfunction and oxidative stress [21]. Specifically, angiotensin II has been shown to increase interleukin-6 expression in the vascular wall, which reduces the bioavailability of nitric oxide and stimulates endothelial expression of adhesion molecules [22]. As such, we hypothesize that CKD patients with greater baseline neurohormonal activation may derive an enhanced anti-inflammatory benefit from ACE-I/ARB therapy. Nevertheless, this hypothesis requires further validation. Particularly, we did not observe lower PCAT attenuation among ACE-I/ARB users in our subgroup with blood pressure ≥130/80 mmHg, even though these patients may have heightened RAAS activity contributing to their uncontrolled hypertension.

The latest guidelines emphasize ACE-I or ARB treatments in patients with diabetic kidney disease [23]. In the RENAAL study, there was a trend towards a lower incidence of MI in patients with T2DM and nephropathy who were treated with losartan [24]. In an observational study of patients who experienced an acute myocardial infarction from the SWEDEHEART registry, treatment with ACE-/ARB yielded lower incidence of recurrent MI in patients with GFRs between 30 and 60 mL/min but not in patients with GFRs above 60 mL/min [25]. We had a limited number of patients with GFR < 60 mL/min (*n* = 24), as medical providers would likely have elected for alternative ischemic evaluation modalities that do not require intravenous contrast in patients with more advanced kidney dysfunction. We observed a trend towards lower PCAT attenuation in patients on RAAS inhibitors for this particular subgroup (*p* = 0.061). However, this finding needs to be further assessed on a larger-scale population. We were also unable to fully account for the role of microalbuminuria, as only 27% of patients had these data available, which is considered a limitation of the study.

We observed a higher proportion of high-intensity statin use in patients on RAAS inhibitors (44.3% vs. 23.8%; *p* = 0.001). This trend is in concordance with recent guidelines advocating for aggressive lipid lowering with high-intensity statin therapy in patients with T2DM and hypertension [26,27]. As high-intensity statins have been shown to possess pleiotropic effects that can stabilize coronary plaques [12,14], aggressive uptitration of these medications should be emphasized in this high-risk cohort. In our multivariate linear regression model, we found that statin intensity was independently associated with lower PCAT attenuation. Furthermore, in our subgroup analysis of patients already on high-intensity statins, we saw no difference in PCAT attenuation between ACE-I/ARB users and their counterparts. As such, we hypothesize that high-intensity statins likely possess a greater protective effect against vascular inflammation than ACE-I or ARB.

The number of patients on tissue-specific ACE-I is low (*n* = 4) in our study. Hoshida et al. showed an increase in ACE activity in culprit lesions from acute coronary syndrome through the analysis of coronary plaques obtained by coronary atherectomy [28]. Ramipril exerts greater tissue activity than other ACE-Is, and the HOPE trial demonstrated lower atherosclerotic events with ramipril in patients with vascular disease or diabetes plus one additional atherosclerotic risk factor [29]. Quinapril, another tissue-specific ACE-I, has previously been shown to improve endothelial dysfunction in normotensive patients [30]. However, it has not been proven that tissue-specific ACE-Is are superior to serum ACE-Is in lowering major adverse cardiovascular outcomes. Further studies are needed to determine whether tissue-specific ACE-I would yield a greater anti-inflammatory effect on coronary plaques.

In addition to ACE-Is and ARBs, the other classes of antihypertensive therapies were not associated with lower coronary inflammation on CCTA. Thus far, no study has been conducted to evaluate how these drug therapies influence coronary plaque progression and inflammation on CCTA. Historically, beta blockers were the standard of care in medical therapy for coronary artery disease. However, recent studies showed a lack of long-term clinical benefits in patients who previously experienced acute myocardial infarction and had preserved left ventricular ejection fraction [31]. Furthermore, calcium channel blockers, aldosterone receptor antagonists, and thiazides have not been shown to yield specific benefits in preventing future myocardial infarction [32,33]. As such, it is plausible that these treatments are not associated with lower coronary inflammation on CCTA.

Our study has several limitations. It is a single-centered, retrospective observational analysis and falls short of the robust methodology of a randomized, prospective clinical trial. We performed propensity score matching, but there are likely residual confounding factors that were not addressed in the analysis. Specifically, the impacts of various hypoglycemic agents, such as SGLT-2 inhibitors, and lipid-lowering therapies (e.g., ezetimibe and proprotein convertase subtilisin/kexin type 9 inhibitors) on PCAT attenuation have not been well-studied, and these factors are not accounted for in our analysis. We were also unable to verify medication compliance beyond clinical documentation. As some studies have demonstrated an association between poor glycemic control and medication non-adherence [34,35], we excluded patients with HbA1C > 9.0%, which does limit the generalizability of our results. Furthermore, we only included patients with a BMI ≤ 35, as patients with larger body habitus tend to have increased adipocyte size. This confounding morphology shifts PCAT radiodensity toward more negative values, which can misrepresent the underlying coronary inflammation [7]. Additionally, while we recorded blood pressure readings in the three clinic visit encounters preceding the CCTA, we were unable to confirm whether proper blood pressure monitoring techniques were used. It should also be noted that the PCAT assessment was carried out by only one cardiovascular imaging specialist. However, there is good interobserver variability with PCAT attenuation measurements, with a prior study noting an intraclass correlation coefficient of 0.996 [36]. We also eliminated CCTAs with poor imaging quality that could skew the assessment.

Although our study findings are more applicable for T2DM patients with a milder degree of renal involvement, they do suggest that ACE-I and ARB therapy may be linked to reduced coronary inflammation in patients with GFR < 90 mL/min. Nevertheless, further analyses are warranted to assess the anti-inflammatory potential of these classes of drugs in a population with a broader spectrum of renal failure. Alternative imaging modalities that do not require intravenous contrast enhancement, such as positron emission tomography, may serve better for this purpose. Finally, only 16% of our study cohort were found to have severe, obstructive coronary artery stenoses that warrant intervention after CCTA. While we did not observe a trend, further studies are warranted to assess the relationship between RAAS inhibitor treatment and vascular inflammation in patients with more advanced coronary artery disease.

## 5. Conclusions

In our cohort of T2DM patients with coronary atherosclerosis, we did not observe an association between treatment with ACE-I or ARB and lower coronary inflammation on CCTA. Such a potential relationship may exist in patients with reduced GFRs, but large-scale, prospective studies are needed to explore these findings in a wider spectrum of chronic kidney disease.

## Figures and Tables

**Table 1:** Baseline characteristics of the two treatment groups based on whether they are on treatment with angiotensin-converting enzyme inhibitors or angiotensin receptor blockers (ACE-I/ARB vs. non-ACE-I/ARB). The ACE-I/ARB group had a higher proportion of patients with a history of hypertension and hyperlipidemia and more patients on high-intensity statins. Statistically significant differences are highlighted in black. Acronyms: BMI, body mass index; CAC, coronary artery calcification; HbA1C, hemoglobin A1C; CT, computed tomography; GLP-1, glucagon-like peptide; SGLT-2, sodium-glucose cotransporter 2; PCAT, pericoronary adipose tissue attenuation; HU, Hounsfield unit.

Variables	Non-ACE-I/ARB (*n* = 101)	ACE-I/ARB (*n* = 122)	*p-* Value
Age (years)	64.0 ± 8.9	65.6 ± 8.7	0.164
BMI (kg/m^2^)	27.7 ± 4.0	28.5 ± 4.1	0.136
CAC score	518.8 ± 781.3	759.5 ± 1026.1	0.057
HbA1C (%)	6.7 ± 0.9	6.8 ± 0.8	0.519
Glomerular filtration rate (mL/min)	87.9 ± 19.0	84.3 ± 17.4	0.148
Creatinine (mg/dL)	0.90 ± 0.28	0.91 ± 0.24	0.803
Sex (male)	74.3%	64.8%	0.146
Race			
Caucasians	35.6%	32.0%	0.572
East Asians	20.8%	22.1%	0.871
African Americans	4.0%	5.7%	0.758
Hispanics	15.8%	22.1%	0.306
South Asians	15.8%	13.1%	0.571
Pacific Islanders	4.0%	3.3%	1
Others	4.0%	1.6%	0.414
**Hypertension**	**75.2%**	**97.5%**	**<0.001**
**Hyperlipidemia**	**82.2%**	**92.6%**	**0.023**
Current smoker	5.0%	5.7%	1
Microvascular complications	40.6%	47.5%	0.344
Coronary interventions required	14.9%	17.2%	0.716
Indications for CT			
Chest pain or anginal equivalent	57.4%	67.2%	0.164
**Preoperative evaluation**	**21.8%**	**9.8%**	**0.015**
Abnormal cardiac testing	13.9%	13.1%	1
Cardiomyopathy evaluation	6.9%	4.1%	0.385
**Other**	**0.0%**	**5.7%**	**0.017**
Medications			
Metformin	89.1%	95.1%	0.128
GLP-1 receptor agonist	13.9%	26.2%	0.204
SGLT-2 inhibitors	18.8%	18.9%	0.368
Insulin	22.0%	15.6%	0.228
Aspirin	36.6%	44.3%	0.275
**High-intensity statins**	**23.8%**	**44.3%**	**0.001**
PCAT attenuation (HU)	−71.6 ± 8.0	−73.5 ± 7.3	0.073

**Table 2:** Multivariate analysis of clinical characteristics for the prediction of pericoronary adipose tissue (PCAT) attenuation. Male sex and statin intensity were found to be independent predictors and were incorporated in the propensity score matching and multivariate linear regression analyses. * Given the right-skewed distribution, the coronary artery calcium (CAC) score was log-transformed using the natural logarithm.

		95% Confidence Interval		
Variables	Beta Coefficient	Lower Bound	Upper Bound	*p* -Value
Age	−0.081	−0.196	0.035	0.304
**Male sex**	**2.655**	**0.443**	**4.867**	**0.019**
**Statin intensity**	**−1.708**	**−0.312**	**−0.297**	**0.018**
Hyperlipidemia	0.302	−2.927	3.531	0.854
Hypertension	−1.265	−4.329	1.798	0.416
Presence of obstructive stenoses	1.659	−1.119	4.437	0.240
CAC score *	−0.369	−0.933	0.195	0.198
Current smoking status	−1.845	−6.268	2.578	0.412

**Table 3:** Comparison of baseline characteristics of patients on angiotensin-converting enzyme inhibitors or angiotensin receptor blockers (ACE-I/ARB) against those not on therapy (non-ACE-I/ARB) after propensity score matching. No statistically significant differences were noted. Acronyms: CAC, coronary artery calcification; HbA1C, hemoglobin A1C; CT, computed tomography; GLP-1, glucagon-like peptide; SGLT-2, sodium-glucose cotransporter 2; PCAT, pericoronary adipose tissue; HU, Hounsfield unit.

Variables	Non-ACE-I/ARB (*n* = 73)	ACE-I/ARB (*n* = 73)	*p* -Value
Age (years)	64.3 ± 9.4	64.7 ± 8.9	0.767
BMI (kg/m^2^)	27.8 ± 3.8	27.8 ± 4.0	0.983
CAC score	589.7 ± 881.1	568.4 ± 960.8	0.899
HbA1C (%)	6.7 ± 0.9	6.8 ± 0.8	0.787
Glomerular filtration rate (mL/min)	86.1 ± 19.5	84.8 ± 17.8	0.686
Creatinine (mg/dL)	0.92 ± 0.30	0.92 ± 0.25	0.992
Sex (male)	72.6%	71.2%	1
Race			
Caucasians	35.6%	26.0%	0.282
East Asians	21.9%	21.9%	1
African Americans	2.7%	6.8%	0.442
Hispanics	15.1%	28.8%	0.071
South Asians	13.7%	15.1%	1
Pacific Islanders	5.5%	0.0%	0.120
Others	5.5%	1.4%	0.366
Hypertension	95.9%	95.9%	1
Hyperlipidemia	90.4%	89.0%	1
Current smoker	6.8%	2.7%	0.442
Microvascular complications	42.5%	45.2%	0.868
Coronary interventions required	16.4%	17.8%	1
Indications for CT			
Chest pain or anginal equivalent	60.3%	69.9%	0.298
Preoperative evaluation	17.8%	11.0%	0.346
Abnormal cardiac testing	13.7%	12.3%	1
Cardiomyopathy evaluation	8.2%	2.7%	0.275
Other	0.0%	4.1%	0.245
Medications			
Metformin	89.0%	94.5%	0.367
GLP-1 receptor agonist	15.1%	17.8%	0.824
SGLT-2 inhibitors	21.9%	28.8%	0.447
Insulin	24.7%	15.1%	0.213
Aspirin	42.5%	49.3%	0.507
High-intensity statins	26.0%	37.0%	0.212
PCAT attenuation (HU)	−71.7 ± 8.1	−72.1 ± 7.5	0.722

**Table 4:** Summary of PCAT attenuation for three common antihypertensive classes: dihydropyridine calcium channel blockers (DH-CCBs), beta blockers, and thiazides. Comparisons were made with multivariate linear regression analysis. None of these therapies was independently linked to lower coronary inflammation on CT.

Classes of Medications	Not on Medication	On Medication	*p* -Value
DH-CCB	−72.3 ± 7.8 (*n* = 179)	−74.0 ± 6.9 (*n* = 44)	0.502
Beta blockers	−72.0 ± 7.2 (*n* = 168)	−74.5 ± 8.6 (*n* = 55)	0.104
Thiazides	−72.4 ± 7.8 (*n* = 189)	−73.8 ± 6.8 (*n* = 34)	0.728

## Data Availability

The datasets presented in this article are not readily available because they are part of an ongoing study and contain private patient information that cannot be shared.

## Supplementary Materials

The following supporting information can be downloaded at: https://www.mdpi.com/article/10.3390/biomedicines14061268/s1, Table S1: Comparison of baseline characteristics of patients on maximal doses of angiotensin-converting enzyme inhibitors or angiotensin receptor blockers (ACE-I/ARB) against those not on therapy (non-ACE-I/ARB) after propensity score matching. No statistically significant differences were noted. Acronyms: CAC, coronary artery calcification; HbA1C, hemoglobin A1C; CT, computed tomography; GLP-1, glucagon-like peptide; SGLT-2, sodium-glucose cotransporter 2; PCAT, pericoronary adipose tissue attenuation; HU, Hounsfield unit.

## References

[1] Shih C.J., Chu H., Ou S.M., Chen Y.T. (2015). Comparative effectiveness of angiotensin-converting enzyme inhibitors and angiotensin II receptor blockers on major adverse cardiac events in patients with newly diagnosed type 2 diabetes: A nationwide study. Int. J. Cardiol..

[2] Lv X., Zhang Y., Niu Y., Song Q., Zhao Q. (2018). Comparison of angiotensin-converting enzyme inhibitors and angiotensin II receptor blockers on cardiovascular outcomes in hypertensive patients with type 2 diabetes mellitus: A PRISMA-compliant systematic review and meta-analysis. Medicine.

[3] Griendling K.K., Ushio-Fukai M., Lassègue B., Alexander R.W. (1997). Angiotensin II signaling in vascular smooth muscle. New concepts. Hypertension.

[4] Brasier A.R., Recinos A., Eledrisi M.S. (2002). Vascular inflammation and the renin-angiotensin system. Arterioscler. Thromb. Vasc. Biol..

[5] Li W., Li J., Hao P., Chen W., Meng X., Li H., Zhang Y., Zhang C., Yang J. (2016). Imbalance between angiotensin II and angiotensin-(1-7) in human coronary atherosclerosis. J. Renin-Angiotensin-Aldosterone Syst..

[6] Awad K., Zaki M.M., Mohammed M., Lewek J., Lavie C.J., Banach M., Lipid and Blood Pressure Meta-analysis Collaboration Group (2022). Effect of the Renin-Angiotensin System Inhibitors on Inflammatory Markers: A Systematic Review and Meta-analysis of Randomized Controlled Trials. Mayo Clin. Proc..

[7] Tan N., Dey D., Marwick T.H., Nerlekar N. (2023). Pericoronary Adipose Tissue as a Marker of Cardiovascular Risk: JACC Review Topic of the Week. J. Am. Coll. Cardiol..

[8] Tzolos E., Williams M.C., McElhinney P., Lin A., Grodecki K., Flores Tomasino G., Cadet S., Kwiecinski J., Doris M., Adamson P.D. (2022). Pericoronary Adipose Tissue Attenuation, Low-Attenuation Plaque Burden, and 5-Year Risk of Myocardial Infarction. JACC Cardiovasc. Imaging.

[9] Nedosugova L.V., Markina Y.V., Bochkareva L.A., Kuzina I.A., Petunina N.A., Yudina I.Y., Kirichenko T.V. (2022). Inflammatory Mechanisms of Diabetes and Its Vascular Complications. Biomedicines.

[10] Antonopoulos A.S., Angelopoulos A., Papanikolaou P., Simantiris S., Oikonomou E.K., Vamvakaris K., Koumpoura A., Farmaki M., Trivella M., Vlachopoulos C. (2022). Biomarkers of Vascular Inflammation for Cardiovascular Risk Prognostication: A Meta-Analysis. JACC Cardiovasc. Imaging.

[11] Yu Y., Ding X., Yu L., Dai X., Wang Y., Zhang J. (2022). Increased coronary pericoronary adipose tissue attenuation in diabetic patients compared to non-diabetic controls: A propensity score matching analysis. J. Cardiovasc. Comput. Tomogr..

[12] Mátyás B.B., Benedek I., Raț N., Blîndu E., Parajkó Z., Mihăilă T., Benedek T. (2024). Assessing the Impact of Long-Term High-Dose Statin Treatment on Pericoronary Inflammation and Plaque Distribution-A Comprehensive Coronary CTA Follow-Up Study. Int. J. Mol. Sci..

[13] Li Y., Yao W., Wang T., Yang Q., Song K., Zhang F., Wang F., Dang Y. (2024). Association of semaglutide treatment with coronary artery inflammation in type 2 diabetes mellitus patients: A retrospective study based on pericoronary adipose tissue attenuation. Cardiovasc. Diabetol..

[14] Wu B., Nieman K., Sandoval R. (2025). High-intensity statin therapy is associated with reduced coronary inflammation on CT in patients with type 2 diabetes mellitus. Diabetes Obes. Metab..

[15] Zhang Y., Ding X., Hua B., Liu Q., Chen H., Zhao X.Q., Li W., Li H. (2020). Real-world use of ACEI/ARB in diabetic hypertensive patients before the initial diagnosis of obstructive coronary artery disease: Patient characteristics and long-term follow-up outcome. J. Transl. Med..

[16] Pitt B., O’Neill B., Feldman R., Ferrari R., Schwartz L., Mudra H., Bass T., Pepine C., Texter M., Haber H. (2001). The QUinapril Ischemic Event Trial (QUIET): Evaluation of chronic ACE inhibitor therapy in patients with ischemic heart disease and preserved left ventricular function. Am. J. Cardiol..

[17] Hirohata A., Yamamoto K., Miyoshi T., Hatanaka K., Hirohata S., Yamawaki H., Komatsubara I., Murakami M., Hirose E., Sato S. (2010). Impact of olmesartan on progression of coronary atherosclerosis a serial volumetric intravascular ultrasound analysis from the OLIVUS (impact of OLmesarten on progression of coronary atherosclerosis: Evaluation by intravascular ultrasound) trial. J. Am. Coll. Cardiol..

[18] Williams C., Han D., Takagi H., Fordyce C.B., Sellers S., Blanke P., Lin F.Y., Shaw L.J., Lee S.E., Andreini D. (2023). Effects of renin-angiotensin-aldosterone-system inhibitors on coronary atherosclerotic plaques: The PARADIGM registry. Atherosclerosis.

[19] Olechnowicz-Tietz S., Gluba A., Paradowska A., Banach M., Rysz J. (2013). The risk of atherosclerosis in patients with chronic kidney disease. Int. Urol. Nephrol..

[20] Ma K., Gao W., Xu H., Liang W., Ma G. (2022). Role and Mechanism of the Renin-Angiotensin-Aldosterone System in the Onset and Development of Cardiorenal Syndrome. J. Renin-Angiotensin-Aldosterone Syst..

[21] Pacurari M., Kafoury R., Tchounwou P.B., Ndebele K. (2014). The Renin-Angiotensin-aldosterone system in vascular inflammation and remodeling. Int. J. Inflam..

[22] Didion S.P. (2017). Cellular and Oxidative Mechanisms Associated with Interleukin-6 Signaling in the Vasculature. Int. J. Mol. Sci..

[23] Virani S.S., Newby L.K., Arnold S.V., Bittner V., Brewer L.C., Demeter S.H., Dixon D.L., Fearon W.F., Hess B., Johnson H.M. (2023). 2023 AHA/ACC/ACCP/ASPC/NLA/PCNA Guideline for the Management of Patients With Chronic Coronary Disease: A Report of the American Heart Association/American College of Cardiology Joint Committee on Clinical Practice Guidelines. J. Am. Coll. Cardiol..

[24] Brenner B.M., Cooper M.E., de Zeeuw D., Keane W.F., Mitch W.E., Parving H.H., Remuzzi G., Snapinn S.M., Zhang Z., Shahinfar S. (2001). Effects of losartan on renal and cardiovascular outcomes in patients with type 2 diabetes and nephropathy. N. Engl. J. Med..

[25] Evans M., Carrero J.J., Szummer K., Åkerblom A., Edfors R., Spaak J., Jacobson S.H., Andell P., Lindhagen L., Jernberg T. (2016). Angiotensin-Converting Enzyme Inhibitors and Angiotensin Receptor Blockers in Myocardial Infarction Patients with Renal Dysfunction. J. Am. Coll. Cardiol..

[26] American Diabetes Association Professional Practice Committee (2024). 10. Cardiovascular Disease and Risk Management: Standards of Care in Diabetes-2024. Diabetes Care.

[27] Arnett D.K., Blumenthal R.S., Albert M.A., Buroker A.B., Goldberger Z.D., Hahn E.J., Himmelfarb C.D., Khera A., Lloyd-Jones D., McEvoy J.W. (2019). 2019 ACC/AHA Guideline on the Primary Prevention of Cardiovascular Disease: A Report of the American College of Cardiology/American Heart Association Task Force on Clinical Practice Guidelines. J. Am. Coll. Cardiol..

[28] Hoshida S., Kato J., Nishino M., Egami Y., Takeda T., Kawabata M., Tanouchi J., Yamada Y., Kamada T. (2001). Increased angiotensin-converting enzyme activity in coronary artery specimens from patients with acute coronary syndrome. Circulation.

[29] The Heart Outcomes Prevention Evaluation Study Investigators (2000). Effects of an angiotensin-converting-enzyme inhibitor, ramipril, on cardiovascular events in high-risk patients. N Engl. J. Med..

[30] Mancini G.B., Henry G.C., Macaya C., O’Neill B.J., Pucillo A.L., Carere R.G., Wargovich T.J., Mudra H., Lüscher T.F., Klibaner M.I. (1996). Angiotensin-converting enzyme inhibition with quinapril improves endothelial vasomotor dysfunction in patients with coronary artery disease. The TREND (Trial on Reversing ENdothelial Dysfunction) Study. Circulation.

[31] Gomes R.A.F., Furtado L.C.C., Montenegro M.V., Filho D.C.S. (2025). Beta-blockers in post-myocardial infarction with preserved ejection fraction: Systematic review and meta-analysis. Cardiovasc. Diagn. Ther..

[32] Jeffers B.W., Robbins J., Bhambri R. (2017). Efficacy of Calcium Channel Blockers Versus Other Classes of Antihypertensive Medication in the Treatment of Hypertensive Patients With Previous Stroke and/or Coronary Artery Disease: A Systematic Review and Meta-Analysis. Am. J. Ther..

[33] Reinhart M., Puil L., Salzwedel D.M., Wright J.M. (2023). First-line diuretics versus other classes of antihypertensive drugs for hypertension. Cochrane Database Syst. Rev..

[34] Feldman B.S., Cohen-Stavi C.J., Leibowitz M., Hoshen M.B., Singer S.R., Bitterman H., Lieberman N., Balicer R.D. (2014). Defining the role of medication adherence in poor glycemic control among a general adult population with diabetes. PLoS ONE.

[35] Adamek K.E., Ramadurai D., Gunzburger E., Plomondon M.E., Ho P.M., Raghavan S. (2019). Association of Diabetes Mellitus Status and Glycemic Control With Secondary Prevention Medication Adherence After Acute Myocardial Infarction. J. Am. Heart Assoc..

[36] Tzolos E., McElhinney P., Williams M.C., Cadet S., Dweck M.R., Berman D.S., Slomka P.J., Newby D.E., Dey D. (2021). Repeatability of quantitative pericoronary adipose tissue attenuation and coronary plaque burden from coronary CT angiography. J. Cardiovasc. Comput. Tomogr..

