# Supplementary material for: Analysis of Pericoronary Adipose Tissue Attenuation in Patients with Type 2 Diabetes Mellitus on Angiotensin-Converting Enzyme Inhibitors and Angiotensin Receptor Blockers: A Propensity-Score-Matched Observational Study
Source: Biomedicines. 2026 Jun 2;14(6):1268. doi: 10.3390/biomedicines14061268 (PMC13296852; doi:10.3390/biomedicines14061268)
Supplement: Supplementary file 1 [file biomedicines-14-01268-s001.zip › biomedicines-4285183-supplementary.pdf]

Variables	Non-ACE-I/ARB (n=44)	ACE-I/ARB (n=44)	p-value
Age (years)	65.9±8.8	65.5±8.5	0.806
BMI (kg/m^2)	27.9±3.6	28.7±3.6	0.287
CAC score	759.5±1072.3	654.9±821.7	0.652
HbA1C (%)	6.7±0.83	6.8±0.87	0.804
Glomerular filtration rate (mL/min)	84.7±19.3	82.4±17.5	0.57
Creatinine (mg/dL)	0.9±0.28	0.93±0.24	0.83
Sex (male)	68.2%	61.4%	0.656
**Race**			
Caucasians	31.8%	38.6%	0.656
East Asians	25.0%	20.5%	0.8
African Americans	2.3%	6.8%	0.616
Hispanics	22.7%	18.2%	0.792
South Asians	9.1%	13.6%	0.739
Pacific Islanders	2.3%	0.0%	1
Others	6.8%	2.3%	0.616
Hypertension	97.7%	97.7%	1
Hyperlipidemia	93.2%	93.2%	1
Current smoker	4.5%	6.8%	1
Microvascular complications	40.9%	47.7%	0.668
Coronary interventions required	15.9%	15.9%	1
Indications for CT			
Chest pain or anginal equivalent	61.4%	65.9%	0.825
Preoperative evaluation	15.9%	2.3%	0.058
Abnormal cardiac testing	11.4%	20.5%	0.383
Cardiomyopathy evaluation	11.4%	2.3%	0.202
Other	0.0%	9.1%	0.116
Medications			
Metformin	86.4%	97.7%	0.11
GLP-1 receptor agonist	13.6%	18.2%	0.772
SGLT-2 inhibitors	27.3%	29.5%	1
Insulin	29.5%	11.4%	0.062
Aspirin	43.2%	52.3%	0.522
High-intensity statins	40.9%	45.5%	0.83
PCAT attenuation (HU)	-73.8±7.9	-74.0±7.1	0.891

Table S1. Comparison of baseline characteristics of patients on maximal doses of angiotensin-converting enzyme inhibitors or angiotensin receptor blockers (ACE-I/ARB) against those not on therapy (non-ACE-I/ARB) after propensity score matching. No statistically significant differences were noted. Acronyms: CAC, coronary artery calcification; HbA1C, hemoglobin A1C; CT, computed tomography; GLP-1, glucagon-like peptide; SGLT-2, sodium-glucose cotransporter 2; PCAT, pericoronary adipose tissue attenuation; HU, Hounsfield unit.
